# Development and pilot testing of a prostate cancer polygenic risk report

**DOI:** 10.1186/s12920-026-02416-4

**Published:** 2026-07-30

**Authors:** Julia N. Griffin, Morgan E. Danowski, Haley L. Gerety, Katherine Lafferty, Marla L. Clayman, Ashley A. Antwi, Mercedes G. Bertero, Charles A. Brunette, Chris Gillespie, Louise Guentert, Niall J. Lennon, Julian Martin, Lauren S. Patel, Jason L. Vassy

**Affiliations:** 1 Boston University Genetic Counseling Program, Boston, MA USA; 2 VA Boston Healthcare System, Boston, MA USA; 3 Broad Clinical Labs, Broad Institute of MIT and Harvard, Burlington, MA USA; 4 Center for Health Optimization and Implementation Research (CHOIR), VA Bedford Healthcare System, Bedford, MA USA; 5 Department of Population and Quantitative Health Sciences, UMass Chan Medical School, Worcester, MA USA; 6 University of Washington, Seattle, WA USA; 7 Harvard Medical School, Boston, MA USA; 8 Boston University School of Medicine, Boston, MA USA; 9 Mass General Brigham, Boston, MA USA

**Keywords:** Polygenic risk scores (PRS), Prostate cancer screening, Risk communication, Genomics, Qualitative research

## Abstract

**Background:**

Polygenic risk scores (PRS) are increasingly being incorporated into clinical care, yet optimal strategies for communicating PRS results to patients and clinicians remain undefined. Effective report design is critical to ensure comprehension and appropriate use, particularly for complex conditions such as prostate cancer where screening decisions are nuanced. We developed and pilot tested patient-facing materials to communicate integrated polygenic and monogenic risk for prostate cancer in the context of a randomized clinical trial.

**Methods:**

We designed a summary report and accompanying Frequently Asked Questions (FAQ) page to communicate prostate cancer genetic risk within the Prostate Cancer, Genetic Risk, and Equitable Screening Study (ProGRESS). Materials were developed through an iterative, multidisciplinary process informed by existing literature on genomic risk communication. We conducted semi-structured interviews with a national sample of eight men eligible for prostate cancer screening to evaluate comprehension, interpretation of visual elements, perceived usefulness, and preferences for improvement. Interviews were transcribed and analyzed using reflexive thematic analysis.

**Results:**

Participants generally found the summary report and FAQ page understandable and visually engaging. Graphical displays of absolute risk, particularly pictograph arrays, facilitated comprehension and helped contextualize risk. Visual cues such as color and bold formatting effectively directed attention to key information, with red coloring perceived as particularly salient for high-risk results. In contrast, more complex visualizations, including bell curves and incidence curves, were frequently misunderstood or not interpreted as intended. Participants expressed a desire for clearer guidance regarding next steps and additional accessible information, suggesting supplementary resources such as hyperlinks or QR codes. Concerns about readability included small font size and high text density.

**Conclusions:**

In this qualitative pilot study, patient-facing materials for communicating prostate cancer PRS were generally well received, with specific design features such as simple visualizations and clear formatting enhancing understanding. Findings highlight the importance of intuitive risk displays and actionable guidance in PRS reporting. These results provide practical insights to inform the design of genomic risk reports as PRS-based prostate cancer screening approaches move toward clinical implementation.

**Trial registration:**

ClinicalTrials.gov NCT05926102; date of registry: July 3, 2023.

**Supplementary Information:**

The online version contains supplementary material available at 10.1186/s12920-026-02416-4.

## Background

Polygenic risk scores (PRS) are emerging as potentially clinically useful tools and are increasingly viewed as ready for clinical implementation [1–6]. As genome-wide association studies (GWAS) have identified novel single nucleotide variants associated with complex common disorders such as coronary artery disease, diabetes mellitus, and several cancers, some PRS can now identify subsets of patients with disease risk equivalent to some monogenic variants, but at a higher population frequency [7–11]. As a result, researchers, clinicians, and other stakeholders increasingly perceive PRS as useful tools for clinical risk stratification and disease prevention and diagnosis, and clinical PRS have now been developed and implemented in multiple research and clinical settings [5, 9, 12–15].

Prostate cancer is one disease where a PRS might have particular clinical utility for risk stratification and early detection. Prostate adenocarcinoma is the most common non-skin malignancy among men and a leading cause of cancer-related mortality [16]. Universal screening for prostate cancer with prostate-specific antigen (PSA) blood testing can reduce prostate cancer mortality but can also overdiagnose indolent disease and lead to unnecessary procedures and treatments [17–20]. As a result, clinical practice guidelines differ on whether to recommend PSA testing and for which patient populations [21–26]. Prostate cancer screening practices vary substantially as a result [20, 27]. At the same time, prostate cancer is one of the most heritable cancers, and an estimated 44% of the familial risk of prostate cancer can be attributed to the hundreds of common genetic variants identified through recent GWAS [28]. A PRS derived from these variants can describe a spectrum of heritable prostate cancer risk, such that as many as one-fifth of men have a PRS indicating an approximately two-fold lifetime risk of prostate cancer [28, 29]. These observations suggest that a prostate cancer PRS might help identify men for whom the benefits of PSA testing (early detection and management) outweigh its risks (unnecessary procedures, overdiagnosis, and overtreatment).

One early step in the clinical implementation of a prostate cancer PRS will be the effective communication of PRS results to patients and their treating clinicians. Genetic testing to understand the risk for certain monogenic hereditary cancer syndromes is well understood [30, 31], and established genetic counseling guidelines inform the way that these results are communicated to patients [32]. This includes the clinical interpretation of variants in established genes associated with prostate cancer risk, such as *BRCA2* and *HOXB13* [30, 33]. While the reporting of monogenic disease risk has established guidelines, such best practices have been conceptually described but not yet firmly established for the more nascent field of PRS generally or the clinical application of prostate cancer specifically [4, 34]. This need becomes more pressing as new evidence emerges that a prostate cancer PRS might enable the detection of a significant proportion of men with prostate cancer for whom current screening methods are inadequate [15]. Additionally, the genetic counseling workforce will be insufficient to meet demand if PRS testing becomes a routine part of all cancer screening decisions [35, 36]. Questions thus remain about how best to present prostate cancer PRS results to patients and clinicians to optimize understanding, including how to balance clarity with completeness, how to display individual numeric risk information, and what contextual information is necessary to support appropriate interpretation and clinical actionability.

We aimed to address some of these questions in the context of the Prostate Cancer, Genetic Risk, and Equitable Screening Study (ProGRESS), a randomized clinical trial of genomics-informed prostate cancer screening [9]. The trial intervention includes a tailored risk report for patients and their primary care clinicians, based on monogenic and PRS results for prostate cancer risk. Before trial participants began receiving their results, we pilot tested these documents among a separate national sample of prostate cancer screening-eligible patients to understand their perceptions, preferences, and needs regarding the information presented on the reports about polygenic results to inform prostate cancer screening.

## Methods

### The prostate cancer, genetic risk, and equitable screening study (ProGRESS)

ProGRESS is a national randomized clinical trial designed to enroll 5,000 prostate cancer screening-eligible men aged 55–69 years [9]. Participants are randomized to either a usual care arm, which receives standard prostate cancer screening information (Supplementary File 1), or a precision screening arm, which receives screening recommendations tailored to individual genetic risk (Supplementary File 2). Genetic risk is determined using both monogenic variants and a polygenic risk model. ProGRESS is approved by the VA Central IRB (Protocol #23–39), and the trial protocol is registered at ClinicalTrials.gov (NCT05926102) as of July 3, 2023.

### Development of PRS report and accompanying materials

Prior to participant recruitment into the ProGRESS trial, a multidisciplinary team of physicians, genetic counselors, clinical laboratorians, and variant curation scientists collaborated over several months to develop the laboratory assay and reporting framework for the precision screening intervention. As previously described [9], the intervention was based on the Prostate CAncer integrated Risk Evaluation (P-CARE) model, which incorporates a prostate cancer polygenic score, family history, and genetic ancestry to stratify prostate cancer risk.

A blended genome–exome (BGE) sequencing assay, which combines low-coverage whole-genome sequencing with high-coverage exome sequencing, was developed to enable simultaneous assessment of both polygenic risk and rare pathogenic variants associated with hereditary prostate cancer. The intervention package includes two laboratory reports: a research-grade PRS report and a Clinical Laboratory Improvement Amendments (CLIA)-certified report for pathogenic or likely pathogenic variants identified in 12 prostate cancer-associated genes. Both reports were designed to align with established clinical genomics reporting standards by providing clear results summaries, categorization of genetic risk, links to guideline-informed recommendations, and supporting technical and interpretive details regarding assay methods, model development, and clinical context [9, 37, 38].

Recognizing the need for accessible results interpretation, the team also developed a patient-facing summary report to synthesize the monogenic and polygenic results and provide tailored screening recommendations. As described in the Results, multiple iterations of this summary report were refined alongside an accompanying Frequently Asked Questions (FAQ) document. Together, these two documents served as patient-accessible cover pages for the more technical laboratory report that followed (full intervention packet in Supplementary File 2).

### ProGRESS interview study

#### Interview design

Following development of the summary report and FAQ document, we conducted a qualitative interview study to evaluate how patients interpret and engage with the PRS communication materials. An interview guide was developed iteratively (Supplemental File 3), to explore 1) familiarity with genetics; 2) familiarity with prostate cancer; and 3) impressions of the ProGRESS summary report and FAQ page, including comprehension of risk information, interpretation of visual elements, perceived usefulness, and suggestions for improvement.

#### Participant recruitment

We recruited a national convenience sample of patients from the ScreenShare Study, a mixed-methods study aimed at improving shared decision-making in precision prostate cancer screening [39, 40]. The ScreenShare Study previously conducted semi-structured interviews with 42 patients at Department of Veterans Affairs (VA) healthcare facilities across 27 U.S. states and territories. Eligible participants were male, aged 40–60 years, receiving VA health care, and had no personal history of prostate cancer. The study employed purposive sampling to ensure diversity by race and ethnicity, geographical region, and prostate cancer screening history, with equal representation of individuals with and without prior PSA testing.

For the present study, ScreenShare participants were eligible if they had completed all study components and consented to future recontact. Participants were invited via email or telephone. Those who completed the interview received a $5 gift card upon interview completion. This study was approved by the VA Central Institutional Review Board as an amendment to the ScreenShare Study protocol (#E23-57).

#### Data collection and analysis

Semi-structured interviews were conducted between November and December 2024 by a two-person team consisting of a genetic counseling student (JG) and a genetic counselor (MD). During the interviews, the summary report and FAQ page were shared on screen, and participants were guided through each section prior to eliciting feedback using the interview guide. Researchers took detailed written notes during the interviews, and all but one were audio-recorded and transcribed. Transcripts were de-identified and manually reviewed to correct transcription errors and ensure data accuracy. The analytic team (JG, MD and HG) used a reflexive thematic analysis approach to analyze the data. Transcripts were systematically reviewed using comparative analysis, and participants’ responses were organized into a matrix to facilitate reflexive thematic analysis [41]. A priori codes informed the initial coding process.

## Results

### Summary report and FAQ page design

**Fig. 1:**
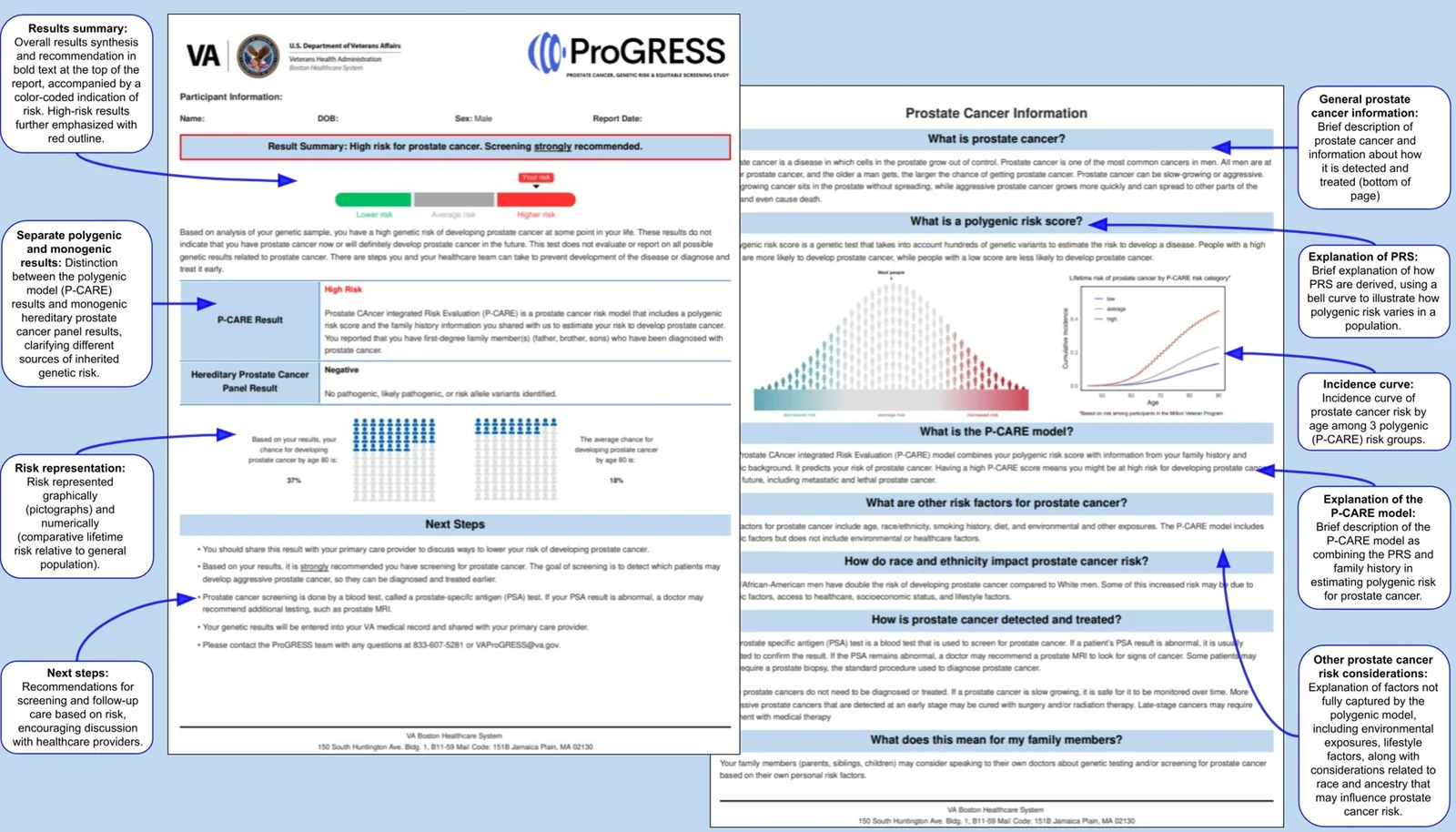
Annotated example of summary report and FAQ page

Figure 1 displays an annotated example of the ProGRESS trial summary report and FAQ page that resulted from the iterative design process described above. The summary report presents integrated P-CARE model results alongside monogenic variant findings to support risk-informed screening decisions. The FAQ page provides foundational information about polygenic scores, prostate cancer risk factors and screening, as well as anticipated patient questions addressed during ProGRESS post-test genetic counseling sessions. Topics include prostate cancer overview, a definition of PRS, the influence of race and ethnicity on prostate cancer, screening methods, and implications for family members. The study genetic counselor reviewed existing publicly available polygenic and monogenic risk reports and research about risk communication to derive content and formatting elements for the ProGRESS documents [42–44], as described below in the Results.

We made the following design decisions based on best practices in risk communication and the existing literature on communicating PRS results [4, 34, 42, 43]. First, the documents use clear, concise, and unambiguous language to communicate the content. Patients and clinicians can find further technical details about the tests on the subsequent PRS and monogenic laboratory reports, but we designed the summary report to be understandable at the 8th-grade reading level. Second, we strategically designed the sequence and formatting of the content to convey meaning [10, 11]. The overall results synthesis and recommendation were presented in bold text at the top of the report, while the separate polygenic P-CARE and monogenic results were presented in separate text boxes further down the page. A section labeled *Next Steps *clearly indicated recommended actions for the patient and his clinician to take. Third, we used multiple graphics to display quantitative risk [42]. The summary report uses part-to-whole pictograph arrays and risk percentages to display lifetime risk of prostate cancer for an individual with a certain PRS category, compared to that in the general population [42]. The FAQ page additionally includes a bell curve to convey the concept of a spectrum of polygenic risk and incidence curves to show cumulative risk across three PRS categories (high, average, and low risk) [11]. Fourth, color was used in both documents to cue the reader to important information and to signify the level of risk. Colors were chosen based on widespread cultural association of green or other cool-toned colors to mean go/less urgent and red or other warm-toned colors to mean stop/more urgent [11].

### Pilot testing among patient participants

**Fig. 2:**
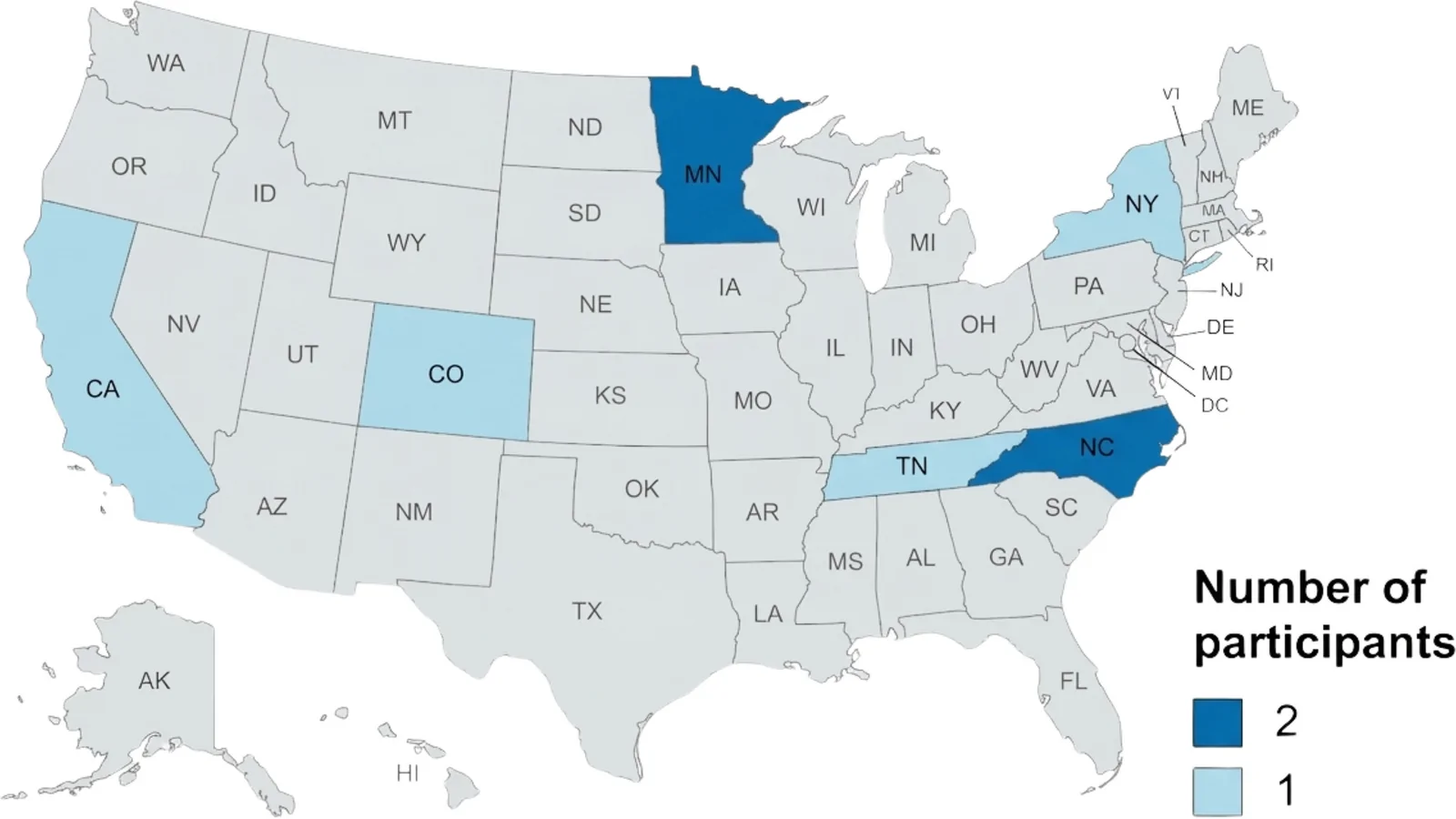
Geographic distribution of interview participants

All ten ScreenShare Study patient participants who consented to being contacted for future research were invited to participate in the qualitative interview study about the ProGRESS trial reports; nine expressed interest, and eight ultimately completed an interview (Fig. 2). These eight participants resided in 6 different U.S. states, reported a range of familiarity with genetic testing, from no prior knowledge to somewhat familiar due to a family history, coursework, preconception testing, or direct-to-consumer testing. Participants similarly reported a range of familiarity with prostate cancer, from no prior knowledge to having a friend or family member with a diagnosis, to working with patients with cancer. Thematic analysis identified three themes related to the patient perceptions of the ProGRESS trial reports: 1) how the visual display of risk information promotes understanding of risk, 2) how formatting elements draw attention to key details of the reports, and 3) suggestions for improvement.

### How the visual display of risk information promotes understanding of risk

Participants frequently used graphical displays of numeric risk information to inform their perceptions of risk. The majority of participants found that the graphics aided comprehension, particularly the pictograph arrays comparing the risk groups. For example, participant 11,202 shared, “it gives a visual picture instead of just a number there. Some people can see a number and not think twice about it, but when you have a visual picture it draws it out and brings your attention to it a little bit more I think." Participants also found the text boxes alongside the pictograph helpful. Some participants, including participant 11,081, commented that they felt 37% was a high and compelling number: *"*the chance of getting prostate cancer at age 80 is 37%. It’s almost 4 out of 10 people. Oh that's quite high.*"* In addition, participant 11,192 stated 37% is a “scary high” number. Conversely, some participants found the 37% lifetime risk to be underwhelming. “37%…it does not seem like a high risk” (Participant 11,200).

While many participants responded favorably to the pictograph, they did not tend to understand or appreciate the bell curve or incidence curve on the FAQ page. Participant 11,192 shared, “I think it says cumulative incidence. I think I’m a fairly smart person but I don’t know what that means.” Another participant also struggled to interpret the incidence curve: “So I think there’s a chance that people would look at this and they’d be like, ‘well this graph just tells me high risk, which makes sense because it’s red and it’s high.’ But the numbers don’t [make sense].” [Participant 11192] One participant expressed that the bell curve was eye-catching, but was unable to interpret the meaning of the graph: *“*Those are bell curves right? A lot of people don’t know how to read those, so they may not look at it. A lot of veterans may not know how to read [bell curves]. The other graph, the cumulative incidence, they would read those better because you have the ages so with higher age is higher risk.” [Participant 11116].

### How formatting elements draw attention to key details of the reports

Participants found the summary report to be helpful, exemplified by Participant 11,200: “You know, from my experience, and a lot of my fellow veterans, we might just look at the first page and not look at anything else.” Participants cited several visual elements that enabled them to quickly identify the main points of the summary report. Participants consistently identified color as one of the most helpful visual cues, both to draw their attention and to understand the risk information conveyed. A majority of the participants responded that color was immediately noticed upon viewing the materials, most of them specifically pointing out red text used for the PRS result. Participant 11,147 shared: *“*I zoomed in on that right away…Because it's circled in red and then the high- the high risk. It got my attention.” Color was appreciated not only in the text but also in the graphs. One participant detailed his interpretation of a graph, sharing:* “*I think there’s a chance that people would look at this and they’d be like, well, this graph just tells me high, high risk, which makes sense because it’s red and it’s high.” [Participant 11192] Many participants shared favorable comments about the overall visual appeal, font choice, and layout of the summary report and expressed appreciation for the FAQ page. *“*How it’s typed out and how it’s colored, underlined, highlighted, bold print…I would say that is important and I would say, I would say it’s a good format*”* [Participant 11202].

### Suggestions for improvement

Despite the perceived strengths of the summary report and FAQ page, the participants provided a number of suggestions for improvement. Two participants expressed that having a written explanation alongside the graphs, particularly the bell curve, would improve their understanding. Another participant suggested adding a “marker” to the bell curve on the FAQ page to indicate an individual’s risk. Participant 11,081 recommended using percentages rather than decimals to label the incidence curves: “That might make it easier for people if they saw a percent: 20%, 30%, 40%, versus 0.1, 0.2.”

Many participants indicated that the small font size and the word count may impact the readability, as the page felt “busy.” Participants cited age-related vision loss as a reason for their own struggle with the font, and noted it may be a concern for other people who are of screening age for prostate cancer. Participant 11,051 shared, "I mean, the older you get, you gotta cater your words to people with glasses, you know,…so maybe the smallest text that you have on the first page should be a little bit bigger if you can." Some participants felt the amount of text on the page should be reduced, to improve readability.

At the same time, many of the participants felt that they would want more information about prostate cancer screening, especially in the event that they were deemed to be high risk. For example, Participant 11,202 noted, “You know, if a person's dealing with this kind of stuff, I don't think there's such a thing as too much…matter of fact, if I was a higher risk, I'd want a whole heck of a lot more.” Participants expressed a desire for more detailed information about next steps and strategies they could implement to mitigate their risk. Participant 11,116 shared, “What is the plan? What do I do? If you have all these stats and tests it’s meaningless. What do I do? Now that you have my information I need direction.” The desire for more information but improved readability led a few of the participants to suggest including a link or a QR code to access additional or supplementary information. They felt this could help to provide more information for those who wanted to “dig more” [Participant 11208], while not overwhelming the page.

## Discussion

We developed and pilot tested patient-facing materials designed to communicate PRS and monogenic results for prostate cancer risk in the context of a prostate cancer screening clinical trial. Interviews with prostate cancer screening-eligible patients suggested they generally found the reports understandable and visually engaging, particularly the graphical displays of risk and visual cues that emphasized key information. Consistent with best practices for risk communication, they found pictograph arrays and estimates of absolute risk estimates to be most informative. Participants also identified areas for improvement, including difficulties interpreting some graphical elements (specifically bell curves), and a desire for clearer guidance regarding clinical next steps. In addition, participants shared concerns about readability; some felt the amount of text on the page should be reduced while others would prefer to receive more information. As genomic risk-stratification approaches move toward clinical implementation, these findings provide early insights into both the design considerations and communication challenges involved in balancing clarity and completeness when presenting prostate cancer PRS results. In our study, we have sought this balance through a detailed technical laboratory report including methods and the names of genes sequenced preceded by two pages of patient-friendly summary and FAQs.

These results contribute to the evolving literature on reporting PRS results to patients. Guidelines exist for the laboratory reporting of monogenic results and include recommendations for standardized approaches to variant interpretation and nomenclature, clinical interpretation for the patient and family members, and the limitations of testing [37, 45]. In contrast, there are no established guidelines for how to report PRS results. In the absence of established standards, a recent practice resource from the National Society of Genetic Counselors highlights that PRS results should be reported using clear visual aids and absolute risk estimates, contextualized within overall genetic and non-genetic risk, and accompanied by explicit communication of their limitations and non-diagnostic nature [4]. Similarly, the American College of Medicine Genetics and Genomics recommends that PRS laboratory reports clearly define the predicted outcome, present both absolute and relative risk with population context, use intuitive visual and numerical formats, explicitly communicate limitations and uncertainty, and avoid deterministic interpretation while situating PRS within overall risk assessment [34]. We designed our prostate cancer PRS report with these considerations in mind, and patient feedback confirmed the value of many of these elements in the prostate cancer context.

In the absence of guidelines, research studies and other implementation projects have generated data on the outcomes of reporting of PRS to patients and clinicians. In user testing in the context of a coronary artery disease PRS report prototype, Brockman and colleagues found that patient understanding, emotional responses, and perceived utility were strongly influenced by report design features, including color, visuals, and explanatory text [43]. The Electronic Medical Records and Genomics (eMERGE) Network has developed and implemented an integrated genome-informed risk assessment report combining PRS with monogenic, family history, and clinical risk information alongside care recommendations [12]; initial user testing found that patients often misinterpreted quantitative risk information on PRS reports and yet still derived a general sense of increased risk and potential actionability, again highlighting that report design strongly influences comprehension, perceived utility, and potential clinical use [46]. In interviews among patients receiving a PRS report for colorectal cancer risk, most reported that visual graphs comparing their risk to population averages promoted understanding and trust in their PRS results [47]. Participants interviewed about a glaucoma PRS report preferred absolute risk visuals (pictographs and pie charts) over relative risk displays (bell curves), which they thought caused confusion and exaggerated perceived risk [48]. Our work extends these findings to prostate cancer specifically, a condition for which PRS may soon play an increasingly important role in risk stratification and screening [15, 49].

We acknowledge the strengths and limitations of the study. The national scope of the study increased the likelihood of receiving a wider range of perspectives and opinions. Qualitative interviews using concrete mock reports allowed for participants to provide nuanced reactions to the PRS materials in a manner not otherwise possible with a more structured interview or survey study. Interview participants were limited to a subset of ScreenShare participants who had completed interviews about the clinical use of PRS in their prostate cancer screening decision. Thus, the participants likely had more familiarity with PRS than the average public, but this familiarity enabled them to be more informed pilot testers of the materials. Reassuringly, our interviews suggested that participants varied in their responses and familiarity with both genetics and prostate cancer, and qualitative research such as the present study can elicit important themes [50, 51]. Finally, this study only elicited the perspectives of patients, not clinicians; additional work from the ScreenShare Study will examine clinician perspectives on PRS in prostate cancer screening. Future research should continue to explore communication preferences for PRS reports, particularly among patients with varying health and digital literacy levels. The ProGRESS Study trial has to date enrolled more than 2,500 men, over 30% of whom are from backgrounds considered underrepresented in biomedical research. That trial will enable examination of the impact of patient characteristics on their psychological and behavioral response to the PRS intervention, including how they understand and act on the information.

## Conclusions

We designed a tailored risk report for PRS and monogenic results for prostate cancer risk specifically, and then demonstrated its usability and comprehensibility among a sample of prostate cancer screening-eligible patients. This work reinforces the need to produce tailored information that adheres closely to best practices, an increasingly difficult task in an era of increasingly complex information. As the appropriate role of PRS in clinical prostate cancer screening is clarified in the near future, this work serves as a model for reporting such results to patients.

## Supplementary Information

Supplementary Material 1.

Supplementary Material 2.

Supplementary Material 3.

## Data Availability

The datasets generated and/or analyzed during the current study are not publicly available because they contain potentially identifiable qualitative interview data from research participants, but de-identified excerpts and additional study materials are available from the corresponding author on reasonable request and with appropriate approvals.

## Abbreviations

BGE: Blended genome–exome
CLIA: Clinical Laboratory Improvement Amendments
FAQ: Frequently Asked Questions
GWAS: Genome-wide association studies
IRB: Institutional Review Board
JSON: JavaScript Object Notation
P-CARE: Prostate CAncer integrated Risk Evaluation
PDF: Portable Document Format
ProGRESS: Prostate Cancer, Genetic Risk, and Equitable Screening Study
PRS: Polygenic risk scores
PSA: Prostate-specific antigen
VA: Department of Veterans Affairs
