# Supplementary material for: Development and pilot testing of a prostate cancer polygenic risk report
Source: BMC Med Genomics. 2026 Jul 30;19:137. doi: 10.1186/s12920-026-02416-4 (PMC13520487; doi:10.1186/s12920-026-02416-4)
Supplement: Supplementary file 1 [file 12920_2026_2416_MOESM1_ESM.pdf]

Supplementary File 1: Usual care prostate cancer screening information

Veterans Health Library

Helping Veterans stay well and well-informed

Prostate Cancer Screening

The prostate is a gland that lies just below the bladder. It wraps around the urethra, which is the tube that carries urine and semen out of the penis. The prostate is part of the male reproductive system.

Problems with the prostate become more common as a man ages. These problems include prostate cancer, a common cancer in men. This cancer can often be cured or controlled, especially if it is found and treated early (when it's still small and only in the prostate). A screening test is used to help find cancer early in people who have no symptoms of cancer. Prostate cancer causes no symptoms in its early stages.

Screening tests may help find prostate cancer before it causes any symptoms. However, studies have shown that all not men should be screened. This is because screening can lead to problems, such as finding cancer that may not need to be treated. This can lead to stress.

It can also lead to harms from:

- False positive results that need more testing
- Possible prostate biopsies
- Having surgery, chemotherapy, or radiation that has no benefit, but instead causes more harm
- Treatment complications such as pain, bleeding, infection, incontinence (not being able to hold urine) and erectile dysfunction
- Major side effects from treatment that may not be needed

Most experts agree that men should talk with their health care providers about the pros and cons of screening before it’s done. And they should make a personal choice about whether or not to have the screening.

What is prostate cancer?

Prostate cancer is an uncontrolled growth of changed (mutated) cells. These cells don't look, act, or work the way they should. Prostate cancer starts in the prostate. But over time, it can grow and spread throughout the body.

Risk factors for prostate cancer

Things that can increase a man’s chance of prostate cancer are called risk factors. These include:

- Age. As you grow older, your risk of developing prostate cancer increases.
- Family history. If your father or brother has had prostate cancer, your risk is higher.

VA Central IRB
Effective Date: October 11, 2024

- Race. African-American men are more likely than other men to develop prostate cancer. They are also more likely to die of prostate cancer than other men with this disease. The reasons for this are not clear.
- Agent Orange exposure. Men who have been exposed to Agent Orange may also be at higher risk for prostate cancer.

Screening for cancer

Screening for prostate cancer may help check if you have cancer before it causes problems. Screening tests often include a prostate specific antigen (PSA) blood test. If you are between ages 55 and 69, talk with your health care provider about the pros and cons of screening so you can make a personal choice about whether or not to have it done.

Before your screening test

Tell your health care provider about:

- Any health problems you have
- Any family members who have had prostate cancer or other related health problems
- Medicines, herbs, vitamins, and supplements that you take
- Any problems with urination or symptoms of urinary tract infection

PSA test

PSA is a protein made by prostate cells. Your PSA level can be measured with a blood test. A PSA level that is high may be caused by prostate cancer. It might also may be caused by another problem that is not cancer. A lower PSA level means that cancer is less likely.

Many factors can affect PSA levels. These include age, an enlarged prostate, or prostate cancer. Other factors can cause short-term PSA changes, such as a prostate infection, certain medicines, or recent sex. Your health care provider can explain how these factors may affect the timing of the PSA test and your results.

Other testing

A high PSA level doesn’t always mean cancer. More tests may need to be done. After looking at the results of your screening tests, your health care provider may advise other tests. These may include:

- Biopsy. This test takes small pieces of tissue (samples) from the prostate using a thin, hollow needle. An imaging test such as a transrectal ultrasound is used to help guide the needle to the right place in the prostate. The tissue samples are then tested in a lab for cancer cells.
- Imaging tests. If cancer is found, imaging tests might be done to help see if it has spread. Bone scans can check whether cancer has spread to bones. CT and MRI scans can find tumors in bones and soft tissues.

VA Central IRB
Effective Date: October 11, 2024
