# Supplementary material for: Development and pilot testing of a prostate cancer polygenic risk report
Source: BMC Med Genomics. 2026 Jul 30;19:137. doi: 10.1186/s12920-026-02416-4 (PMC13520487; doi:10.1186/s12920-026-02416-4)
Supplement: Supplementary file 2 [file 12920_2026_2416_MOESM2_ESM.pdf]

Supplementary File 2: Full report packet, including patient-facing summary and Frequently Asked Questions (FAQ) document

U.S. Department of Veterans Affairs
Veterans Health Administration
Boston Healthcare System

ProGRESS
PROSTATE CANCER, GENETIC RISK & EQUITABLE SCREENING STUDY

Participant Information:

Name: **DOB:** **Sex:** Male **Report Date:**

Result Summary: High risk for prostate cancer. Screening strongly recommended.

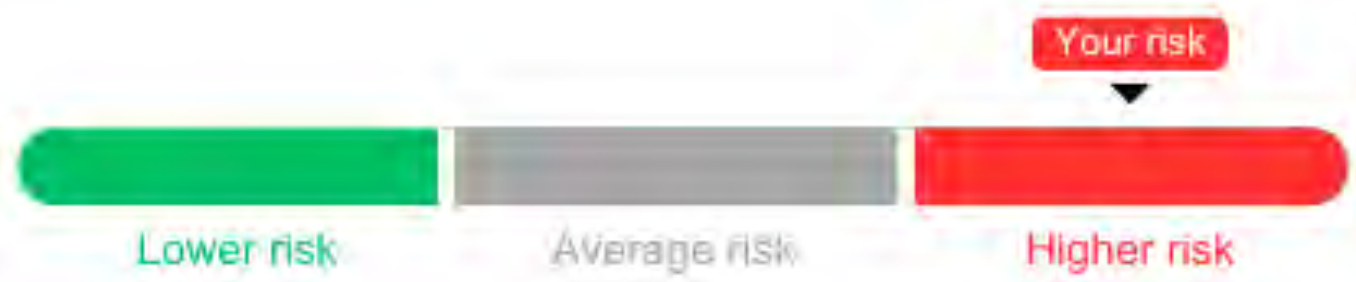

Based on analysis of your genetic sample, you have a high genetic risk of developing prostate cancer at some point in your life. These results do not indicate that you have prostate cancer now or will definitely develop prostate cancer in the future. This test does not evaluate or report on all possible genetic results related to prostate cancer. There are steps you and your healthcare team can take to prevent development of the disease or diagnose and treat it early.

**P-CARE Result**	**High Risk**<br><br>Prostate CAncer integrated Risk Evaluation (P-CARE) is a prostate cancer risk model that includes a polygenic risk score and the family history information you shared with us to estimate your risk to develop prostate cancer. You reported that you have first-degree family member(s) (father, brother, sons) who have been diagnosed with prostate cancer.
**Hereditary Prostate Cancer Panel Result**	**Negative**<br><br>No pathogenic, likely pathogenic, or risk allele variants identified.

Based on your results, your chance for developing prostate cancer by age 80 is:

37%

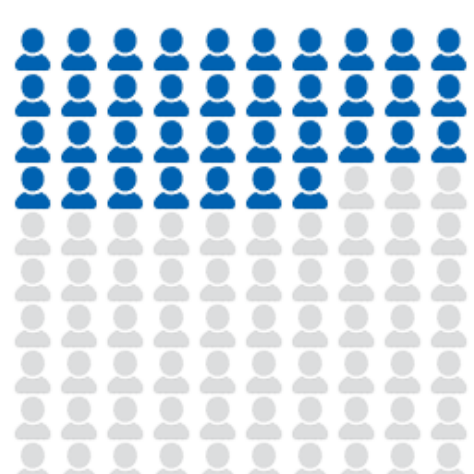

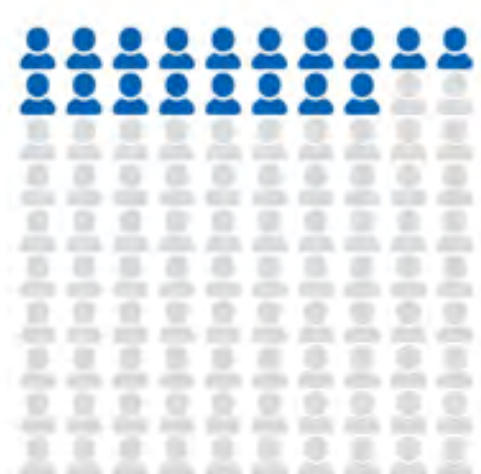

The average chance for developing prostate cancer by age 80 is:

18%

Next Steps

- You should share this result with your primary care provider to discuss ways to lower your risk of developing prostate cancer.
- Based on your results, it is strongly recommended you have screening for prostate cancer. The goal of screening is to detect which patients may develop aggressive prostate cancer, so they can be diagnosed and treated earlier.
- Prostate cancer screening is done by a blood test, called a prostate-specifc antigen (PSA) test. If your PSA result is abnormal, a doctor may recommend additional testing, such as prostate MRI.
- Your genetic results will be entered into your VA medical record and shared with your primary care provider.
- Please contact the ProGRESS team with any questions at 833-607-5281 or VAProGRESS@va.gov.

Prostate Cancer Information

What is prostate cancer?

Prostate cancer is a disease in which cells in the prostate grow out of control. Prostate cancer is one of the most common cancers in men. All men are at risk for prostate cancer, and the older a man gets, the larger the chance of getting prostate cancer. Prostate cancer can be slow-growing or aggressive. Slow-growing cancer sits in the prostate without spreading, while aggressive prostate cancer grows more quickly and can spread to other parts of the body and even cause death.

What is a polygenic risk score?

A polygenic risk score is a genetic test that takes into account hundreds of genetic variants to estimate the risk to develop a disease. People with a high score are more likely to develop prostate cancer, while people with a low score are less likely to develop prostate cancer.

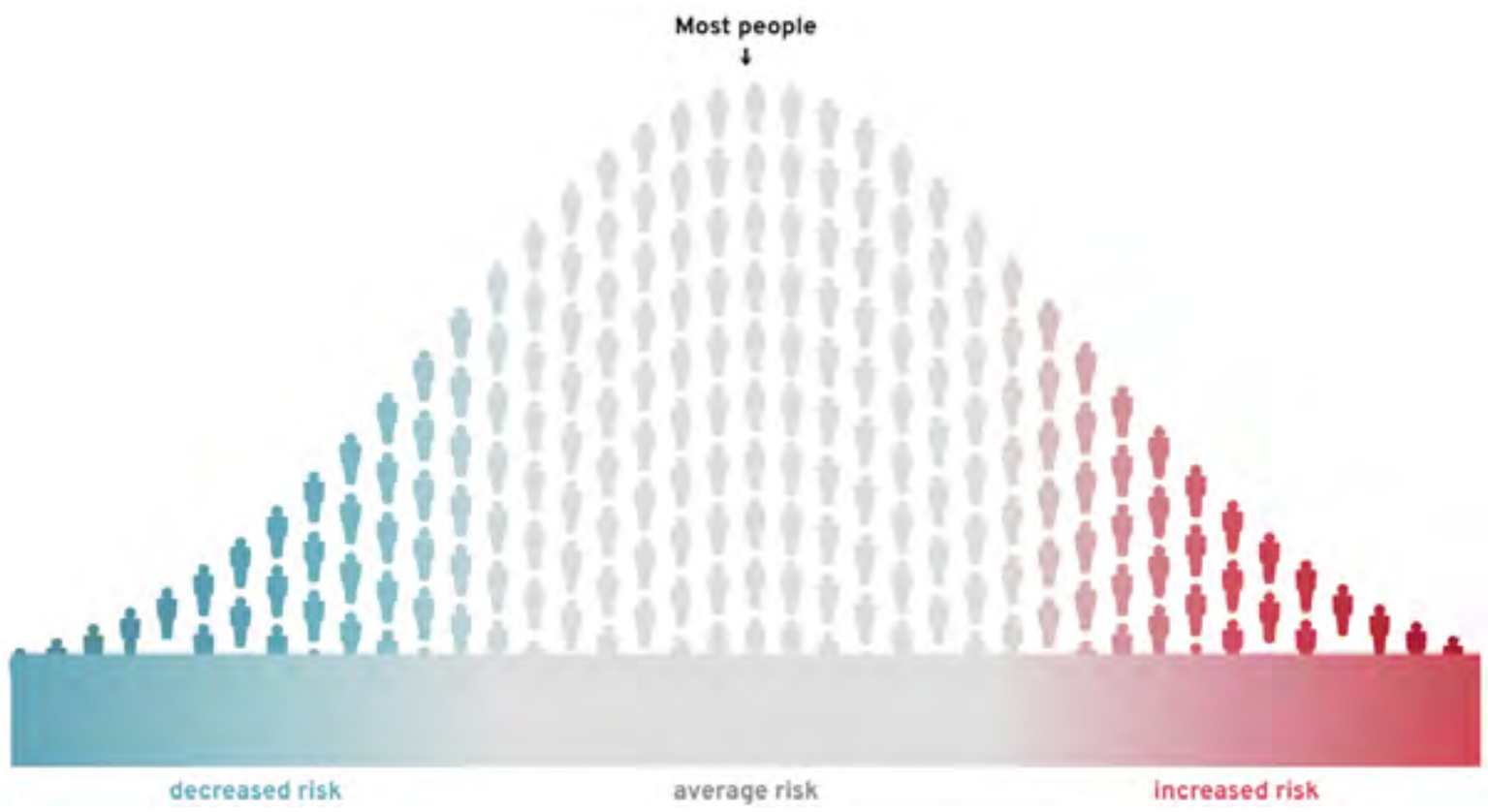

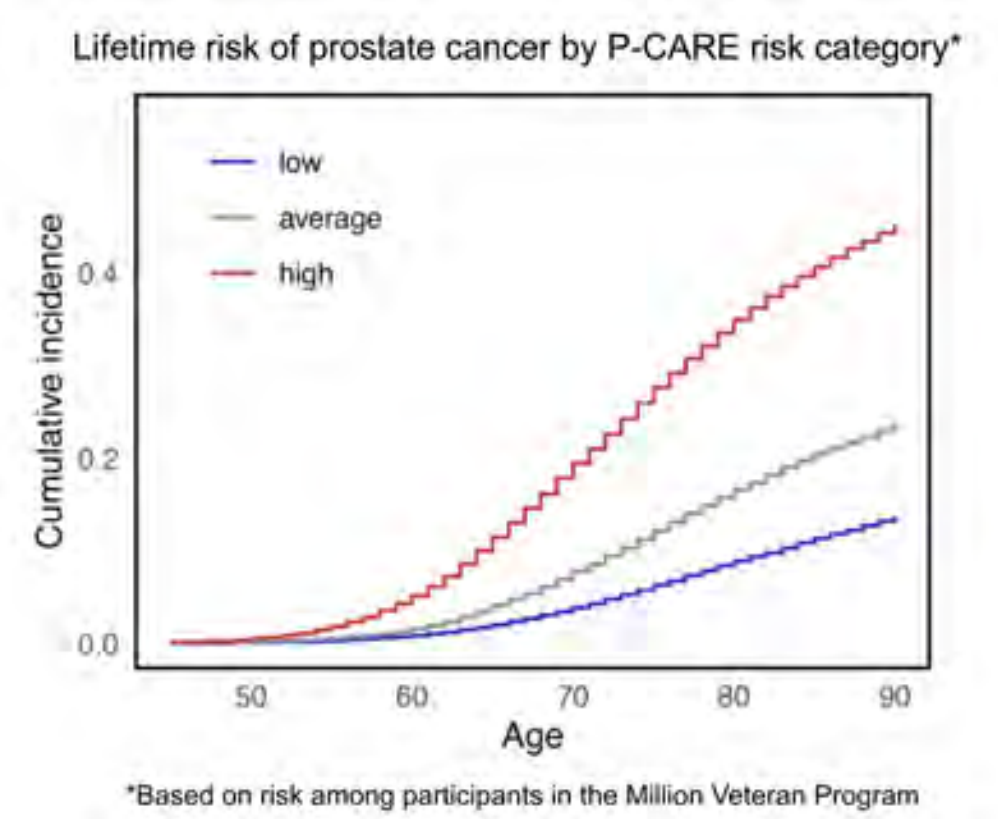

What is the P-CARE model?

The Prostate CAncer integrated Risk Evaluation (P-CARE) model combines your polygenic risk score with information from your family history and genetic background. It predicts your risk of prostate cancer. Having a high P-CARE score means you might be at high risk for developing prostate cancer in the future, including metastatic and lethal prostate cancer.

What are other risk factors for prostate cancer?

Risk factors for prostate cancer include age, race/ethnicity, smoking history, diet, and environmental and other exposures. The P-CARE model includes genetic factors but does not include environmental or healthcare factors.

How do race and ethnicity impact prostate cancer risk?

Black/African-American men have double the risk of developing prostate cancer compared to White men. Some of this increased risk may be due to genetic factors, access to healthcare, socioeconomic status, and lifestyle factors.

How is prostate cancer detected and treated?

The prostate specific antigen (PSA) test is a blood test that is used to screen for prostate cancer. If a patient's PSA result is abnormal, it is usually repeated to confirm the result. If the PSA remains abnormal, a doctor may recommend a prostate MRI to look for signs of cancer. Some patients may then require a prostate biopsy, the standard procedure used to diagnose prostate cancer.

Some prostate cancers do not need to be diagnosed or treated. If a prostate cancer is slow growing, it is safe for it to be monitored over time. More aggressive prostate cancers that are detected at an early stage may be cured with surgery and/or radiation therapy. Late-stage cancers may require treatment with medical therapy

What does this mean for my family members?

Your family members (parents, siblings, children) may consider speaking to their own doctors about genetic testing and/or screening for prostate cancer based on their own personal risk factors.

BROAD CLINICAL LABS

ProGRESS
PROSTATE CANCER, GENETIC RISK & EQUITABLE SCREENING STUDY

Patient	Sample	Order
**Patient Name:**	**Sample ID:**	**Ordering Clinician:** Jason L. Vassy
**DOB:**	**Accession ID:**	**Ordering Clinical Institution:** VA Boston Healthcare System
**Patient ID:**	**Collection Date:**	**Institution Address:** VA Boston Healthcare System, 150 South Huntington Ave, 151B, Boston, MA 02130
	**Received Date:**	
	**Material Type Received:** Saliva	
	**Report Date:**	

Indication for Testing

Hereditary Prostate Cancer Screening

Test Name

Hereditary Prostate Cancer Panel, Broad Institute

Test Result

Negative Result

This test did not identify any pathogenic or likely pathogenic variants in the 12 genes included in this panel that met reporting criteria (see methods and limitations for full gene list and for reporting criteria). Genetic variants of uncertain significance were not included in this report. A negative result reduces, but does not rule out, the risk of genetic conditions tested for in this panel, nor can it rule out genetic conditions not included in this panel.

As part of the ProGRESS study, some individuals may have received polygenic risk assessment in conjunction with this monogenic gene panel test. Please refer to the ProGRESS Study Result Summary for prostate cancer screening recommendations.

Recommendations

Genetic counseling is available to help individuals understand these results and address any questions. You may contact the ProGRESS genetic counselor at VAProGRESS@va.gov. For further assistance in locating nearby genetic counseling services, please contact your provider or find a genetic counselor through the National Society of Genetic Counselors (https://findageneticcounselor.nsgc.org/). For questions about this report and interpretation please contact bcl-support@broadinstitute.org.

Cancer screening recommendations should factor in personal and family history, genetic and non-genetic risk factors, and population-based guidelines. Screening recommendations, including high risk screening, can be found through the National Comprehensive Cancer Network at www.nccn.org. Work with your healthcare team to determine what cancer screening and/or management is best for you.

Individuals who meet genetic testing criteria for hereditary cancer risk based on personal and/or family history may require additional testing. To determine the most appropriate testing recommendations, contact your health care team or high-risk specialists. These results should be interpreted in the context of the patient’s medical and family history. Please note that variant classification may change over time if more information becomes available. Testing limitations are detailed below.

Patient

Patient Name: **DOB:** **Patient ID:**

Test Methodology

Clinical Blended Genome Exome (cBGE) with interpretive analysis restricted to the genes of interest is performed using the following procedures. Genomic DNA is extracted from the submitted specimen and fragmented, adapter ligated, and barcoded to create a single PCR free whole genome. An aliquot from the genome is taken through PCR amplification and exome selection. A blend of the PCR free genome and whole exome was created and sequenced to deliver an optimal coverage balance. Library fragments are sequenced (2x150 base paired end) using Sequencing-By-Synthesis (SBS) chemistry via the Illumina NovaSeq sequencer. Sequencing data are aligned to the GRCh38 assembly after discarding low quality sequences. Illumina's DRAGEN (v4.2.7; Dynamic Read Analysis for GENomics) platform was used for demultiplexing, read mapping, genome alignment, read sorting, duplicate marking, and variant calling. The DRAGEN pipeline generates quality metrics, a CRAM file (compressed BAM file), and a VCF (Variant Call Format) file that contains single nucleotide variants and insertions/deletions (SNVs/indels) with a target region of +/-16 base pairs into the introns. The GATK-gCNV copy number variant calling pipeline uses the alignments from the DRAGEN produced CRAM file to generate CNV calls with a depth based CNV detection algorithm, utilizing a PoN (panel of normals) to adjust for coverage biases that may arise in data generation and processing. The SNVs/indels and CNV VCF files are merged into one concatenated VCF for downstream tertiary analysis.

Specimen data must meet the following thresholds: ≥ 90% of bases at or greater than 20X and mean target coverage ≥ 60X within the exome. Other quality metrics that are reviewed are percent contamination (≤ 2.5%), percent mapped (≥ 75%), and percent callability (≥ 95%). A sex concordance check between reported sex and BGE-predicted sex will be done to confirm sample swaps have not occurred during sample processing.

A concatenated VCF file containing small variants (SNVs/indels) and CNVs is used as input to the Fabric Genomics platform, which utilizes the ACE pipeline for small variant identification, filtering, and classification for variants in the genes of interest (listed below). ACE provides preliminary auto-classification of small variants based on the codable ACMG/AMP sequence variant interpretation evidence codes (Richards et al. 2015. PMID: 25741868). All small variants located in coding and flanking intronic regions of the panel genes are examined. CNVs will be filtered based on quality scoring and non-passing copy number variant calls will not be interpreted nor reported. CNVs involving panel gene(s) will be considered passing and will be reviewed if they meet the following thresholds: QUAL ≥ 50 for duplications, QUAL ≥ 100 for heterozygous deletions, and QUAL ≥ 400 for homozygous deletions. Additional transcript information for panel genes is available upon request and all reportable variants will include transcript information within the variant nomenclature.

Only variants classified as Pathogenic or Likely Pathogenic in genes tested are reported (see below for gene list). Risk alleles, except for the HOXB13 p.Gly84Glu variant, are not reported. Variants of uncertain significance are not reportable in any gene and will not be returned. Copy number variants are reported in the context of their impact to panel genes only (see Test Limitations section below).

Variants are reported using HGVS nomenclature (https://hgvs-nomenclature.org/stable/) and are interpreted and classified according to ACMG/AMP guidelines (Richards, S. et al., 2015. PMID: 25741868). Gene-disease validity is determined based on recommendations from the Clinical Genome Resource and Strande et al. 2017 (PMID: 28552198).

Low confidence reportable sequence small variants (SNVs and small InDels) and copy number variants (CNVs) are orthogonally confirmed at the discretion of the laboratory director.

Methods used for confirmation include but are not limited to: PCR-based analysis, array-based analysis, Sanger sequencing, and long read sequencing. The technology used may vary based on the detection ability of each method for the region/variant of interest.

Genes Evaluated: ATM, BRCA1, BRCA2, CHEK2, EPCAM, HOXB13, MLH1, MSH2, MSH6, PALB2, PMS2, and TP53

Patient

Patient Name: **DOB:** **Patient ID:**

Test Limitations

The coverage of the exome region in the Blended Genome Exome product is lower than standard clinical exome products and therefore likely has reduced sensitivity and includes more regions where variants cannot be called confidently compared to other diagnostic products. In addition, the genome region outside of the exome is at a lower coverage, by design. This region is intended for imputation and downstream processing for GWAS or PRS determination, for example. It is not suitable for standard variant calling. The low pass genome data is not used to determine the presence of a copy number variant or structural variant, nor to determine the breakpoints (size) of a copy number variant or structural variant that is outside of the exome, and they are not called in a VCF file.

Certain variants including short tandem repeats, structural rearrangements (e.g. translocations, inversions, gene conversion events, etc), and large chromosomal events (e.g. aneuploidy) as well as variants in repetitive regions (e.g. homopolymers, centromeres, telomeres, etc), in the mitochondrial genome, and regions with low coverage, mapping quality, and base quality are currently not reliably detected by short-read genomic sequencing and not evaluated as part of this test. Copy number detection from exome data shows decreased sensitivity for smaller events, particularly for deletions or duplications smaller than three exons.

Sequencing data is limited to the tissue source used for DNA extraction. This test has not been validated for low-level mosaicism of less than 15%; however, these findings may still be reported. Additional testing with higher coverage, such as a targeted test or an exome, is recommended to confirm potential mosaic variants. The accuracy of results may be impacted for samples submitted with a hematological malignancy, bone marrow transplant, and/or recent blood transfusion. If there are any concerns about these scenarios, please contact the laboratory immediately.

Variants of uncertain significance are not reported nor are variants that do not meet the guidelines as reportable findings noted above. All interpretations are based on currently available data. However, these interpretations may change as new information becomes available (see Methodology). A new report may be released to the ordering provider if new information generates a reportable update.

Coverage and callability analysis was performed to assess gene regions with technical limitations. All technical performance measures were met for all genes on this panel except for PMS2. This gene showed undercovered regions in exons 13-15; therefore, variants that fall within these exons are not reported as part of this test. In general, technical limitations of this test can be a result of sequencing challenges due to high homology, repetitive or difficult sequences, or genes that contain certain types of pathogenic variants that cannot be detected by this technology. This can impact the ability to accurately call variants and create higher false negative rates in these regions. Please contact the lab for details regarding these specific regions.

Because this panel analysis is performed from the exome sequencing data, it is possible that a copy number event that extends outside a panel gene is detected. This report only includes information about the copy number event's impact on the genes designated on this panel. This analysis does not assess the full extent of a variant outside of a panel gene. Furthermore, resolution of exact breakpoints of copy number events from short read sequencing data may not be possible and additional clinical confirmatory studies are indicated in those cases. This information could impact the predicted pathogenicity of a copy number variant depending on the exact disruption point within the gene.

Regulatory Disclosures

This test was performed by Broad Clinical Laboratories, LLC. (27 Blue Sky Drive, Burlington, MA 01803; CLIA: 22D2055652). BCL, LLC. is authorized under the Clinical Laboratory Improvement Amendments (CLIA) to develop and perform high complexity clinical laboratory testing. This test was developed and its performance characteristics determined by BCL, LLC. The U.S. Food and Drug

Patient

Patient Name: **DOB:** **Patient ID:**

Regulatory Disclosures (CONT.)

Administration (FDA) has not approved or cleared this test; however, FDA clearance or approval is not currently required for clinical use.

Clinical Laboratory Director: Heidi Rehm, PhD, FACMG

bcl-support@broadinstitute.org

CLIA: 22D2055652, CAP: 8707596

Commonwealth of Massachusetts Clinical Laboratory License: 5347

Test results reviewed and approved by:

Diana Toledo PhD, HCLD
Associate Lab Director
Sign-out performed at Location: Code A BCL-support@broadinstitute.org

01/28/2026

Broad IDs

Broad Patient ID:
Broad Family ID:
Broad Sample ID:
Order Number:

U.S. Department of Veterans Affairs
Veterans Health Administration
Boston Healthcare System

ProGRESS P-CARE Report

PATIENT INFORMATION

Patient Name:

Date of Birth:

Age: N/A

Family ID:

Patient ID:

Sample ID:

REFERRING PROVIDER

Provider Name: Jason L Vassy

Referring Facility: VA Boston Healthcare System

Referring Facility Address: VA Boston Healthcare System 150 South Huntington Ave, 151B Boston MA US 02130

Test Performed: Polygenic Risk Evaluation

SPECIMEN

Result Status: Final

Result Code: N/A

Collected Date:

Result Date:

Material Type: Bodily Fluid:Saliva

Material Source: Bodily Fluid:Saliva

Results Summary

In this patient the Prostate CAncer integrated Risk Evaluation (P-CARE) result for prostate cancer was determined to be:

HIGH RISK FOR PROSTATE CANCER based on combined genetic score and family history.

*See detailed results for a description for how this risk was determined.

Detailed Results

Prostate Cancer Risk: HIGH RISK

1. Prostate cancer is a disease of abnormal cell growth in the prostate. It is the most common non-skin cancer in men and the 2nd leading cause of cancer death in men. All men are at risk for prostate cancer; however, the risks for developing and dying from prostate cancer increase with age, with the highest incidence observed in men over 65. Prevalence of prostate cancer varies among different ethnic and racial groups, with the highest prevalence observed in African-American men. Prostate cancer is often asymptomatic in its early stages but can be associated with bone pain if it spreads to other parts of the body.
2. A high risk P-CARE result for prostate cancer was found in this individual. P-CARE is a prostate cancer risk model that combines a polygenic score, genetic ancestry, and family history of prostate cancer. A high P-CARE result is associated with a 1.5-fold hazard (50% increased hazard) for developing metastatic prostate cancer relative median P-CARE values (40th-60th percentile). A low P-CARE result is associated with a 0.75-fold hazard (25% lower hazard) for developing metastatic prostate cancer relative to median P-CARE values (40th-60th percentile). All other P-CARE results are categorized as average risk.
3. Factors including monogenic disease risk, family history, and other clinical measures can have an impact on the individual's overall (absolute) risk and should be considered. This result should be interpreted in the context of the patient's medical history, family history, and racial/ethnic background.

Laboratory Comments

N/A

Methodology

Blended genome-exome

Data is generated from blending whole exome sequencing data at a mean target depth of >85X with whole genome sequencing data at a mean target depth of 2.5X coverage. The input into the cBGE process is genomic DNA that was extracted from either blood, saliva, or buccal swabs. gDNA was enzymatically fragmented, adapter ligated, and barcoded to create a single PCR free whole genome. An aliquot from the genome was taken through PCR amplification and exome selection. A blend of the PCR free genome and whole exome was created and sequenced to deliver an optimal coverage balance. Library fragments were sequenced (2x150 base paired end) using Sequencing-By-Synthesis (SBS) chemistry on the Illumina NovaSeq X Plus sequencer. Sequencing data were aligned to the GRCh38 assembly after discarding low quality sequences. Illumina's DRAGEN (Dynamic Read Analysis for GENomics) platform was used for demultiplexing, read mapping, genome alignment, read sorting, duplicate marking, and variant calling. The DRAGEN pipeline generates the quality metric that meets the following thresholds: ≥ 90% of exome bases at or greater than 20X, genome mean coverage ≥ 1X, and mean target coverage ≥ 60X. Other quality metrics that are reviewed are percent contamination (≤ 2.5%), percent mapped (≥ 75%), and percent exome callability (≥ 95%). The DRAGEN pipeline also generates a CRAM file (compressed BAM file), and a hard-filtered VCF (Variant Call Format) file that contains SNPs/indels (single nucleotide variants and small insertions/deletions) with a target region of +/-16 base pairs into the introns. Detailed methods are available upon request. Assay limitations for Germline Analysis are below. The intention of the WES data is to identify rare variants in the exome. The low pass WGS is able to provide low confidence calls for most positions in the genome, and combined with an imputation method (ex. GLIMPSE2) can provide higher confidence haplotype calls to then calculate Polygenic Risk Scores (PRS) for different complex diseases. Millions more SNVs in the sample were determined through imputation using data from the 1000 Genomes Project as a reference panel (Khera et al. 2018).

P-CARE Model

The Prostate CAncer integrated Risk Evaluation (P-CARE) model is an updated version of an earlier model described in Pagadala JNCI 2023 and was developed among 646,991 participants of the Million Veteran Program (18.9% African, 70.6% European, 8.8% American, 1.6% East Asian) and validated among 18,457 participants in the PRACTICAL Consortium. Validation information may be insufficient or not available for populations of other descent. Variants considered for inclusion in the PHS included variants from a prior PHS, variants identified as prostate cancer susceptibility loci in multi-ancestry genome-wide association studies, variants identified as susceptibility loci for benign elevation of prostate-specific antigen (PSA) or benign prostatic hypertrophy (BPH), variants identified as prostate cancer susceptibility loci in men of African ancestry in a genome-wide meta-analysis, and variants identified as susceptibility loci for prostate cancer in a genome-wide multi-ancestry meta-analysis. The final P-CARE model consists of a polygenic hazard score of 601 variants (coefficient 1.097), patient-reported history of prostate cancer in at least one first-degree family member (coefficient 0.4889), and the first 2 principal components of genetic ancestry derived from 2,309 ancestry-informative markers (coefficients -0.0028 and 0.001). In the Million Veteran Program, a P-CARE result above the 80th percentile was associated with a hazard ratio of 2.75 (2.66-2.84), 2.78 (2.54 - 2.99), and 2.59 (2.22 - 2.97) for any prostate cancer, clinically significant prostate cancer, and metastatic prostate cancer, respectively, compared to the median. A P-CARE result below the 20th percentile was associated with a hazard ratio of 0.43 (0.42 - 0.45), 0.43 (0.40 - 0.46), and 0.45 (0.41 - 0.52) for any prostate cancer, clinically significant prostate cancer, and metastatic prostate cancer, respectively, compared to the median.

Limitations

- A P-CARE result is neither deterministic nor diagnostic. Some people with a 'high risk' result will never develop prostate cancer, while others with a 'low risk' result still have a risk of developing prostate cancer. Therefore, this test is not intended to diagnose a disease or to make surgical or pharmacological intervention decisions. This test does not tell a patient anything about their current state of health and should not substitute for regular visits to the doctor. Any

diagnostic or treatment decisions should be based on additional testing and/or other information that is managed by a healthcare provider.

- This test detects genetic variants that are predefined. This test does not evaluate or report on all possible genetic variation related to a given disease. The test used for the P-CARE model will not detect novel or rare genetic variants and will not rule out the presence of these additional genetic variants related to a disease.
- The predefined list of genetic variants tested in this assay may be different from another institution or company, therefore genetic risk calculations and polygenic scores may differ if compared across different institutions or companies.
- The hazard ratio (HR) listed for each condition does not take into account other factors that may play a role in a patient's overall risk of developing a disease (e.g monogenic risk of developing the disease or environmental and lifestyle risk factors).
- Receiving genetic test results may induce patient anxiety. Patients should speak to their doctor or healthcare provider regarding these test results and potential implications for their health and lifestyle decisions. If you have questions regarding your test results, you can contact the ProGRESS clinical study team using the instructions provided to you during the consent process.
- Although the polygenic score and P-CARE model have been developed to maximize the ability to predict risk in all ancestries, the availability of population reference data means that the score is currently most accurate for those with European genetic ancestry. Due to this population limitation, as well as assay performance and processing issues, some patients may not receive a polygenic risk calculation for every condition listed.

Regulatory Disclosures

This test was performed by Broad Clinical Laboratories, LLC. (27 Blue Sky Drive, Burlington, MA 01803; CLIA: 22D2055652). BCL, LLC. is authorized under the Clinical Laboratory Improvement Amendments (CLIA) to develop and perform high complexity clinical laboratory testing. This test was developed and its performance characteristics determined by BCL, LLC. The U.S. Food and Drug Administration (FDA) has not approved or cleared this test; however, FDA clearance or approval is not currently required for clinical use.

Clinical Laboratory Director: Heidi Rehm, PhD, FACMG
bcl-support@broadinstitute.org
CLIA: 22D2055652, CAP: 8707596
Commonwealth of Massachusetts Clinical Laboratory License: 5347

References

Centers for Disease Control and Prevention, “Prostate Cancer Basics.” 22 Feb. 2024.

Conti DV, Darst BF, Moss LC, et al. Trans-ancestry genome-wide association meta-analysis of prostate cancer identifies new susceptibility loci and informs genetic risk prediction. Nat Genet. 2021 Jan;53(1):65-75. doi: 10.1038/s41588-020-00748-0. Epub 2021 Jan 4. Erratum in: Nat Genet. 2021 Jan 20: PMID: 33398198

Khera AV, Chaffin M, Aragam KG, Haas ME, Roselli C, Choi SH, Natarajan P, Lander ES, Lubitz SA, Ellinor PT, Kathiresan S. Genome-wide polygenic scores for common diseases identify individuals with risk equivalent to monogenic mutations. Nat Genet. 2018 Sep;50(9):1219-1224. doi: 10.1038/s41588-018-0183-z. Epub 2018 Aug 13. PMID: 30104762; PMCID: PMC6128408.

Pagadala MS, Lynch J, Karunamuni R, Alba PR, Lee KM, Agiri FY, Anglin T, Carter H, Gaziano JM, Jasuja GK, Deka R, Rose BS, Panizzon MS, Hauger RL, Seibert TM. Polygenic risk of any, metastatic, and fatal prostate cancer in the Million Veteran Program. J Natl Cancer Inst. 2023 Feb 8;115(2):190-199. doi: 10.1093/jnci/djac199. PMID: 36305680; PMCID: PMC9905969.

Rawla P. Epidemiology of Prostate Cancer. World J Oncol. 2019 Apr;10(2):63-89. doi: 10.14740/wjon1191. Epub 2019 Apr 20. PMID: 31068988; PMCID: PMC6497009.

Testing performed under the direction of Heidi L. Rehm, PhD, FACMG
