# Supplementary material for: Development and pilot testing of a prostate cancer polygenic risk report
Source: BMC Med Genomics. 2026 Jul 30;19:137. doi: 10.1186/s12920-026-02416-4 (PMC13520487; doi:10.1186/s12920-026-02416-4)
Supplement: Supplementary file 3 [file 12920_2026_2416_MOESM3_ESM.pdf]

Supplementary File 3: Interview guide

Interview Guide Draft

INTERVIEWER USES THESE PROMPTS TO EXPLORE PARTICIPANT'S PERCEPTIONS OF THE PRESENTATION OF INFORMATION IN A PROSTATE-CANCER SPECIFIC POLYGENIC RISK SCORE (PRS) REPORT BUT MAY ADAPT THE PROMPTS BASED ON THE SPECIFIC RESPONSES.

INTERVIEWER PROVIDES AN INTRODUCTION, FOR EXAMPLE:

Thank you for agreeing to participate in this interview. Today I would like to ask you a few questions about the results of a specific type of genetic test called a polygenic risk score. A polygenic risk score is a genetic test that takes into account hundreds of genetic variants to estimate the risk to develop a disease.

This interview will focus specifically on prostate cancer. Prostate cancer is a disease in which cells in the prostate grow out of control. Prostate cancer is one of the most common cancers in men. All men are at risk for prostate cancer, and the older a man gets, the larger the chance of getting prostate cancer.

INTERVIEWER MAY CONSIDER ASKING THE FOLLOWING QUESTIONS:

1. Can you tell me about any experiences you have with genetics?
2. Can you tell me about any experiences you have with prostate cancer?
3. What factors do you think would affect your chance to develop a health condition like prostate cancer?

[INTERVIEWER SHOWS PRS REPORT] We would like you to tell us what the risk presentations mean to you, and whether or not they are easy to understand. We know people don't always think about risk in terms of numbers and graphs, so we would like to know how you interpret these presentations of prostate cancer risk.

4. What was the first thing you noticed about the report?
    a. Walk me through the order of how you read it
5. What is the main thing the report is trying to tell you?
6. What was the easiest to understand/most helpful part?
7. What was the most challenging to understand/least helpful part?
8. What is your understanding about this person's risk to develop prostate cancer?
    a. What helped you understand this person's risk?
9. What do the figures tell you?
10. What would you change about this report and why?

INTERVIEWER MAY CONSIDER ASKING THE FOLLOWING QUESTIONS, AS TIME ALLOWS:

11. How would you describe a polygenic risk score?
12. What do you think the difference between high risk, average risk, and low risk are?
13. What does the color coding tell you?
14. What questions do you have about the figures?
15. Do you think you can reduce your risk to develop prostate cancer
    a. If so, how?
16. Who would you prefer to review these results with? (e.g. your PCP, the research team, etc.)
17. How do you think people might feel after receiving this result?
18. Anything else you would like to share about your experience viewing this report?
